# Primary care referrals of patients with potentially serious diseases to the emergency department or a quick diagnosis unit: a cross-sectional retrospective study

**DOI:** 10.1186/1471-2296-15-75

**Published:** 2014-04-28

**Authors:** Xavier Bosch, Ona Escoda, David Nicolás, Emmanuel Coloma, Sara Fernández, Antonio Coca, Alfonso López-Soto

**Affiliations:** 1 Department of Internal Medicine, Hospital Clínic, Institut d’Investigacions Biomèdiques August Pi i Sunyer (IDIBAPS), University of Barcelona, Villarroel 170, Barcelona 08036, Spain

**Keywords:** Quick diagnosis unit, Emergency department, Primary care, Referrals, Anemia, Cancer

## Abstract

**Background:**

In Spain, primary healthcare (PHC) referrals for diagnostic procedures are subject to long waiting-times, and physicians and patients often use the emergency department (ED) as a shortcut. We aimed to determine whether patients evaluated at a hospital outpatient quick diagnosis unit (QDU) who were referred to ED from 12 PHC centers could have been directly referred to QDU, thus avoiding ED visits. As a secondary objective, we determined the proportion of QDU patients who might have been evaluated in a less rapid, non-QDU setting.

**Methods:**

We carried out a cross-sectional retrospective cohort study of patients with potentially serious conditions attended by the QDU from December 2007 to December 2012. We established 2 groups of patients: 1) patients referred from PHC to QDU (PHC-QDU group) and 2) patients referred from PHC to ED, then to QDU (PHC-ED-QDU group). Two observers assessed the appropriateness/inappropriateness of each referral using a scoring system. The interobserver agreement was assessed by calculating the kappa index. Multivariate logistic regression analysis was performed to identify the factors associated with the dependent variable ‘ED referral’.

**Results:**

We evaluated 1186 PHC-QDU and 1004 PHC-ED-QDU patients and estimated that 93.1% of PHC-ED-QDU patients might have been directly referred to QDU. In contrast, 96% of PHC-QDU patients were found to be appropriately referred to QDU first. The agreement for PHC-QDU referrals (PHC-QDU group) was rated as excellent (ϰ = 0.81), while it was rated as good for PHC-ED referrals (PHC-ED-QDU group) (ϰ = 0.75). The mean waiting-time for the first QDU visit was longer in PHC-QDU (4.8 days) than in PHC-ED-QDU (2.6 days) patients (*P = .001*). On multivariate analysis, anemia (OR 2.87, 95% CI 1.49–4.55, *P < .001*), rectorrhagia (OR 2.18, 95% CI 1.10–3.77, *P = .01*) and febrile syndrome (OR 2.53, 95% CI 1.33-4.12, *P = .002*) were independent factors associated with ED referral. Nearly one-fifth of all QDU patients were found who might have been evaluated in a less rapid, non-QDU setting.

**Conclusions:**

Most PHC-ED-QDU patients might have been directly referred to QDU from PHC, avoiding the inconvenience of the ED visit. A stricter definition of QDU evaluation criteria may be needed to improve and hasten PHC referrals.

## Background

Long waiting times and emergency department (ED) overcrowding are common in countries with public health systems, even when primary healthcare (PHC) systems are strong [1].

Over the last decade, the annual demand for ED care in Spain has grown by > 4% compared with a population rise of 2%, and has often surpassed the growth in ED capacity [2]. In Spain, around 30% of ED consultations are deemed inappropriate, and while some ED have attempted to improve efficiency by redesigning circuits, most of the resulting benefits are lost after a few years [2].

There is evidence that the referral process is often suboptimal, with the consequent impact on patients, costs and the system itself. However, despite inappropriate use, including cases better handled in PHC, not all PHC centers have the facilities and resources to diagnose and treat urgent cases needing same-day care, even when not life-threatening [1,3]. Although some single tests, such as blood testing and chest X-rays, are performed rapidly, other diagnostic studies, including CT scans, magnetic resonance imaging and digestive endoscopies, which are usually carried out at reference hospitals, may take several weeks even when malignancy is suspected.

Spanish physicians and patients frequently use the ED to circumvent the time-consuming process of PHC referrals to a specialist or for diagnostic tests [4], potentially contributing to ED overcrowding and increased hospital admissions. The delays involved in the diagnostic and referral process have led to a search for alternatives, most notably hospital-based quick diagnosis units (QDU) for patients with suspected serious disease such as anemia or cancer [4,5]. Besides avoiding hospitalizations and PHC referrals to ED [6], the QDU model has demonstrated equal efficacy, lower costs and greater patient satisfaction compared with conventional admissions [4-6].

The main objective of this study was to determine whether QDU patients referred to ED from PHC could have been safely referred to QDU from the beginning, thus avoiding ED visits. We also determined the appropriateness of direct QDU referrals from PHC and, as a secondary objective of the study, the proportion of QDU patients who might have been evaluated in a less rapid, non-QDU setting.

## Methods

### Characteristics and functioning of the unit

In the setting of an 865-bed tertiary hospital in Barcelona (Spain) attending a population of 540,000, the outpatient internal medicine QDU assesses patients with suspected serious disease processes who are well enough to attend several appointments for diagnostic tests and QDU visits; therefore, they should ideally be stable clinically, mentally and physically capable of attending such outpatient appointments, in order to undergo quick diagnostic examinations [5,6]. Patients with some pulmonary disorders such as lung nodes are normally evaluated by the respiratory disease day center, although QDU evaluation is not ruled out. Referrals come mainly from the hospital ED and a network of 12 PHC centers after training in the referral criteria, and self-referral is not permitted. Referral by e-mail or fax warrants some control of the appropriateness of the referral.

The unit is run by an internal medicine specialist and a nurse, who are assisted by physicians from other specialties. It has a consulting room and a waiting room for patients and companions, and operates daily. The QDU physician and nurse dedicate 5-hours daily, from Monday to Friday, to QDU work. The QDU work is based on a rapid first visit, followed by preferential arrangement of diagnostic investigations and successive visits until a diagnosis is reached. In particular, in addition to a complete anamnesis and physical examination, all patients have laboratory tests during the first appointment, mainly blood and urine analysis (or stool analysis) and chest X-rays if needed. Blood transfusions are given as required in the daycare center of another hospital department. During the following QDU visits, patients’ outcome is evaluated and results of diagnostic tests are checked over. Further examinations are performed according to the results of previous ones or the clinical course of the disease.

### Study design and population

We performed a cross-sectional retrospective study of patients attended by the QDU who fulfilled previously established QDU referral criteria (Table 1) [6]. To avoid potential biases, patients fulfilling referral criteria who did not complete the QDU evaluation due to hospitalization or death, and patients lost to follow-up were also included. The study was approved by the research ethics committee of the Hospital Clínic of Barcelona.

**Table 1:** **Referral criteria of QDU**[6]

**Criteria**	**Explanation**
Febrile syndrome	Fever of unknown origin and temperature ≥ 38 °C for ≥ 14 days
Unintentional weight loss	Unaccounted for loss of ≥10% of body weight, anorexia, asthenia for ≥ 42 days
Anemia	Hemoglobin level < 9 g/L, with or without symptoms
Chronic diarrhea	Loose stools for ≥ 28 days
Adenopathies and/or palpable masses	-
Unexplained severe abdominal pain	-
Rectorrhagia	-
Jaundice	-
Severe constipation (recent onset)	-
Lung and/or pleural abnormalities	Mainly suggestive of neoplasm. After exclusion of obvious causes including community-acquired pneumonia or residual lesions
Unexplained dyspnea	-
Ascites and/or anasarca	-
Dysphagia	-
Arthritis	Degenerative osteoarthritis excluded
Bone pain with suspicion of bone malignancy	-
Splenomegaly and/or hepatomegaly	No known liver or hematological disease (e.g., chronic myelogenous leukemia)
Hemogram abnormalities suggestive of primary hematological disorder	-
Neurologic disorders (central, spinal and peripheral nervous system)	Cerebrovascular disease, delirium, dementia, movement and sleep disorders, dizziness, vertigo, and neuropathic pain excluded.
	Includes Horner’s syndrome
Monoclonal paraprotein band with or without suspicion of multiple myeloma	-

QDU denotes quick diagnosis unit.

Patients included were categorized in 2 groups: 1) patients referred from PHC to QDU (PHC-QDU) and 2) patients referred from PHC to ED, and then to QDU (PHC-ED-QDU). All patients were attended between December 2007 and December 2012, with some having been enrolled in a former prospective study [6].

All patients underwent a complete diagnostic workup according to the protocols for each condition. Diagnoses were made by the QDU physician according to the International Statistical Classification of Diseases and Related Health Problems (Tenth Revision) before onward referral [7].

During the period March-May 2013, two observers (senior internal medicine residents with at least 4-years’ training in general internal medicine wards and QDU) independently reviewed all medical records to assess individual appropriateness of each referral. We estimated the proportion of PHC-ED-QDU patients who might have been safely referred to QDU first, avoiding the ED, and the proportion in whom the ED was an appropriate choice. In PHC-QDU patients, we estimated the proportion in whom the QDU was an appropriate choice and the proportion of patients for whom attending the ED first would have been more appropriate.

A scoring system for objectively assessing the appropriateness of referrals to QDU and ED was devised using key criteria. Firstly, the clinical stability/severity of the patient condition at the time of referral to QDU or ED was itemized in a standardized form and scored using a system based on the Acute Physiology and Chronic Health Evaluation II (APACHE II) severity of disease classification system, originally designed to determine the disease severity of adult patients admitted to intensive care units [8]. Briefly, we scored physiological variables including blood pressure, heart rate, temperature, respiratory rate, oxygen saturation or, when available, partial oxygen pressure, arterial pH (when available), Glasgow coma score, serum creatinine, sodium, potassium, hematocrit and white blood cell count. In addition, we entered the presence of chronic organ insufficiency (liver, renal, cardiovascular and respiratory) or an immunocompromised state and a chronic health score was determined. In line with the APACHE II scoring system [8], we added together age points plus total physiology score plus chronic health points to make the total APACHE II score. Secondly, clinically active presence of the following manifestations at the time of referral was also tabulated and scored: dyspnea, decompensated heart failure, pain and pain severity (according to the 0–10 Numeric Rating Scale [9], calculated at the first QDU visit), anemic syndrome, worsening anemia at PHC (i.e., PHC follow-up showing decreasing hemoglobin and hematocrit levels; see below hospital access to PHC electronic health records), vomiting, diarrhea, external bleeding, fever, decreased oral intake, dysphagia, dehydration, edemas and/or anasarca, single or multiple dyselectrolytemia, and malnutrition laboratory findings. Thirdly, the QDU physician assessment of patient comorbidities and health-related quality of life/functional status using the Charlson score (used to assess the burden of chronic illness) [10] and the SF-12 survey administered at the first QDU visit (a multipurpose short form survey with 12 questions that assesses physical functioning, role limitations due to emotional, health problems, and mental health as well as health concepts like bodily pain, general health, vitality, and social functioning [11]) was also introduced in the worksheet. For patients aged ≥ 70 years, we entered the functional capacity for basic activities of daily living (systematically measured in the QDU using the modified, 10-item Barthel index [12]).

In a separate analysis, medical records from all PHC-QDU and PHC-ED-QDU patients were reviewed by the QDU attending physician (a consultant internist with 28 years’ clinical experience in general internal medicine and emergency medicine) in an attempt to estimate the potential proportion of patients who might have been studied in a less rapid, non-QDU setting such as the hospital outpatient clinics or the own PHC centers. For this purpose, data of each patient during his or her PHC evaluation, before QDU or ED referral, were carefully assessed. Besides reviewing the patient information contained in the PHC formal referral sheets, we searched and examined the electronic health records at the different PHC centers, which can be accessed from the hospital system. Since 2007, electronic medical records of patients evaluated at the hospital and its PHC centers can be visualized and shared by their corresponding physicians. We read PHC physicians’ notes at successive patients appointments to understand the reason for their referral decision. For each patient, PHC information was itemized and scored using spreadsheets standardized for each reason for consultation (i.e., febrile syndrome, anemia, unintentional weight loss and so on). Briefly, in addition to any recorded details about patient medical history (e.g., number of comorbidities), general health and functional status, quality of life or degree of dependence that could have influenced referral decisions, we scored relevant symptoms of the current process under investigation, abnormal findings on physical examination (e.g. characteristics of peripheral adenopathies in patients with such a reason for consultation), disease duration, number of PHC and ED visits (and any hospitalization) in the last 6 months, results from preliminary diagnostic tests (e.g. fecal occult blood tests in patients whose reason for consultation was anemia), and treatments that could have induced referral decisions (e.g., treatment with iron or long-lasting use of anticoagulants for cardiovascular diseases in patients with iron-deficiency anemia). For consistency purposes, all the tabulated PHC information was checked against the QDU and ED (in PHC-ED-QDU patients) information.

### Statistical analysis

Normally distributed continuous variables were expressed as mean and standard deviation and assessed using the student’s t test, and skewed variables were expressed as median and 25% and 75% percentiles and assessed using the Mann–Whitney U non-parametric test. Multivariate logistic regression analysis was performed to identify the factors associated with the dependent variable ‘ED referral’ and the odds ratios (OR) with 95% confidence intervals (CI) for the factors were calculated. A *P* value smaller than .05 was considered statistically significant. Interobserver agreement was assessed by tabulating the distribution of each observer’s results, noting the percentage agreement and calculating the kappa index with their CI. We used the following guidelines to interpret kappa statistic: <0.20, poor agreement; 0.20–0.40; fair agreement; 0.41–0.60, moderate agreement; 0.61–0.80, good agreement; and 0.81–1.00, excellent agreement [13]. All analyses were done using the SAS v.9.1 statistical package (SAS, Cary, North Carolina).

## Results

### Patients evaluated

A total of 2190 patients were evaluated, including 1186 PHC-QDU patients and 1004 PHC-ED-QDU patients. Of PHC-QDU patients, 4 were hospitalized, 2 died and 3 were lost to follow-up. Of PHC-ED-QDU patients, 3 were hospitalized, 2 died and 2 were lost to follow-up.

### Patients’ characteristics

General characteristics are shown in Table 2. The wait for the first QDU visit was significantly longer in PHC-QDU patients, with no differences being observed with respect to age, Charlson comorbidity index, visits per patients, time to diagnosis, and onward referral.

**Table 2:** Main characteristics and differences of the two groups of patients

**Variable**	**PHC-QDU patients n = 1186**	**PHC-ED-QDU patients n = 1004**	***P *** **value**
Age (years)	58.8 (16.1), 62 [55;67.7]	56.8 (15.5), 60.3 [53.8;66]	*NS*
Female	608 (51.3)	527 (52.5)	
Male	578 (48.7)	477 (47.5)	
Main reason for consultation	Unintentional weight loss	Anemia	
Waiting time for 1st QDU visit (days)*	2-8 (4.8)	2-4 (2.6)	*.001*
Charlson com in. (score)	1.3 (0.9), 1.1 [0.9;1.5]	1.2 (0.8), 0.9 [0.7;1.3]	*NS*
Main diagnosis	Malignant neoplasm	Benign GI disorder-related iron-deficiency anemia	
Visits per patient (n)	3.2 (1.8), 3 [2.8;3.3]	2.9 (1.6), 2.7 [2.6;3]	*NS*
Time to diagnosis (days)	9.2 (4), 8.6 [7.9;10.7]	8.9 (4), 8.4 [7.8;10.4]	*NS*
*Destination*			
PHC	690 (58.2)	625 (62.2)	*NS*
Outpatients	465 (39.2)	365 (36.4)	*NS*
Palliative care	20 (1.7)	7 (0.7)	*NS*
Other	11 (0.9)	7 (0.7)	*NS*

Data expressed as mean (SD) and median [25th-75th percentiles] or number (percentage).

*Data expressed as range (mean).

PHC denotes primary care; QDU, quick diagnosis unit; ED, emergency department; *NS*, nonsignificant; Charlson com in., Charlson comorbidity index.

### Appropriateness of referrals

Based on the review of medical records and the total score per patient, we estimated that initial ED visits might have been avoided by direct referral to QDU in 93.1% of PHC-ED-QDU patients. Nevertheless, 36 (3.6% of PHC-ED-QDU patients) patients with anemia were appropriately referred to ED vs. 3.3% of patients with the remaining conditions.

Among PHC-QDU patients, 96% were considered to be appropriately referred to QDU. However, 8 (0.7%) patients with anemia (4.1% of PHC-QDU patients with anemia) had a follow-up showing a decreasing hemoglobin level during the QDU evaluation, which prompted blood transfusion or treatment with intravenous iron in all them. While 1 of these patients had to be hospitalized, the remaining 7 continued their QDU study without further events. In addition, 1 PHC-QDU patient with pancreatic cancer who had also been appropriately referred died during the QDU evaluation and postmortem examination revealed massive pulmonary embolism. Referrals were considered inappropriate in 4% of PHC-QDU patients, most notably 1.8% (n = 21) of patients with anemia who should have been referred to ED first (Table 3).

**Table 3:** Number of PHC-QDU patients who should have been referred to the ED and of PHC-ED-QDU patients who should have been directly referred to the QDU according to the reason for consultation

**Reason for consultation**	PHC-QDU patients* n = 1186	**PHC-ED-QDU patients† n = 1004**
Anemia	21 (1.8)	247 (24.6)
Unintentional weight loss	4 (0.3)	119 (11.9)
Febrile syndrome	5 (0.4)	223 (22.2)
Adenopathies and/or palpable masses	0 (0)	37 (3.7)
Lung and/or pleural abnormalities	1 (0.08)	28 (2.8)
Chronic diarrhea	2 (0.2)	20 (2)
Abdominal pain	3 (0.3)	29 (2.9)
Ascites	3 (0.3)	20 (2)
Rectorrhagia	1 (0.08)	160 (15.9)
Jaundice	3 (0.3)	10 (1)
Dysphagia	0 (0)	14 (1.4)
Arthritis	0 (0)	9 (0.9)
Other	5 (0.4)	19 (1.9)
Total	48 (4)	935 (93.1)

Data expressed as number (percentage).

*Patients who should have been referred to the ED first.

†Patients who should have been directly referred to the QDU.

PHC denotes primary care; QDU, quick diagnosis unit; ED, emergency department.

QDU referrals were also considered inappropriate in 4 out of 6 PHC-QDU patients who were eventually hospitalized or died and in 3 out of 5 PHC-ED-QDU patients who were hospitalized or died.

### Potential evaluation in a less rapid, non-QDU setting

Although, as explained, all patients fulfilled QDU referral criteria, the QDU physician estimated that 217 (18.3%) PHC-QDU patients might have been studied in another less rapid, less acute setting. (e.g., outpatient clinic or the own PHC center). In addition, a non-QDU evaluation might have been more appropriate in 19 out of 1004 (1.9%) PHC-ED-QDU patients.

### Interobserver agreement

The agreement of the appropriateness/inappropriateness of patients directly referred from PHC to QDU (PHC-QDU group) was rated as excellent, while it was rated as good for patients referred from PHC to ED (PHC-ED-QDU group) (Table 4).

**Table 4:** Interobserver agreement of the appropriateness/inappropriateness of referrals

**Variable**	**Agreement (%)**	**Unadjusted ϰ**	**CI 95%**
PHC-QDU referral	89.6	0.81	0.77-0.85
n = 1186*			
PHC-ED referral	85.6	0.75	0.71-0.79
n = 1004^†^			

*PHC-QDU group.

^†^PHC-ED-QDU group.

PHC denotes primary care; QDU, quick diagnosis unit; ED, emergency department; CI, confidence interval.

### Reasons for consultation

Table 5 shows the 13 main reasons for consultation and their differences between the 2 groups. Significantly more PHC-ED-QDU patients presented with anemia, febrile syndrome and rectorrhagia than PHC-QDU patients, while significantly more PHC-QDU patients presented with unintentional weight loss than PHC-ED-QDU patients.

**Table 5:** Main reasons for consultation and differences of the two groups of patients

**Reason for consultation**	**PHC-QDU patients n = 1186**	**PHC-ED-QDU patients n = 1004**	***P *** **value**
Anemia	195 (16.4)	283 (28.2)	*.005*
Unintentional weight loss	293 (24.7)	123 (12.2)	*.004*
Febrile syndrome	135 (11.4)	237 (23.6)	*.003*
Adenopathies	107 (9)	25 (2.5)	*NS*
Palpable masses	42 (3.5)	12 (1.2)	*NS*
Lung and/or pleural abnormalities	66 (5.6)	31 (3.1)	*NS*
Chronic diarrhea	52 (4.4)	22 (2.2)	*NS*
Abdominal pain	76 (6.4)	30 (3)	*NS*
Ascites	26 (2.2)	21 (2.1)	*NS*
Rectorrhagia	44 (3.7)	163 (16.2)	*.006*
Jaundice	21 (1.8)	12 (1.2)	*NS*
Dysphagia	24 (2)	14 (1.4)	*NS*
Arthritis	36 (3)	9 (0.9)	*NS*
Total	1117 (94.2)	982 (97.8)	

Data expressed as number (percentage).

PHC denotes primary care; QDU, quick diagnosis unit; ED, emergency department; *NS*, nonsignificant.

### Diagnoses

PHC-QDU patients had a significantly higher prevalence of malignancies compared to PHC-ED-QDU patients. In contrast, PHC-ED-QDU patients had a significantly higher prevalence of non-malignancy-related iron-deficiency anemia, benign colonic disorders and acute viral illness compared to PHC-QDU patients (Table 6).

**Table 6:** Main diagnoses and differences of the two groups of patients

**Diagnosis**	**PHC-QDU patients n = 1186**	**PHC-ED-QDU patients n = 1004**	***P *** **value**
*Malignant neoplasm*	356 (30)	174 (17.3)	*.003*
Colorectal	91 (7.7)	7 (0.7)	*NS*
Lymphoma	83 (7)	32 (3.2)	*NS*
Gastric	23 (1.9)	26 (2.6)	*NS*
Lung	48 (4)	25 (2.5)	*NS*
Pancreatic	53 (4.5)	51 (5.1)	*NS*
Other hematological*	15 (1.3)	11 (1.1)	*NS*
Breast	14 (1.2)	10 (1)	*NS*
Other neoplasms	29 (2.4)	12 (1.2)	*NS*
*Iron-deficiency anemia*	158 (13.3)	254 (25.3)	*.002*
Digestive	120 (75.9)	194 (76.2)	*NS*
Unknown cause	18 (11.4)	31 (12.2)	*NS*
Heavy menstrual bleeding	12 (7.6)	19 (7.5)	*NS*
Other causes	8 (5.1)	10 (4.1)	*NS*
*Multifactorial anemia*	17 (1.4)	6 (0.6)	*NS*
*Megaloblastic anemia*	14 (1.2)	6 (0.6)	*NS*
*Other types of anemia*	6 (0.5)	2 (0.2)	*NS*
*Chronic liver disease*	40 (3.4)	7 (0.7)	*NS*
*Irritable bowel syndrome*	56 (4.7)	15 (1.5)	*NS*
*Inflammatory bowel disease*	15 (1.3)	6 (0.6)	*NS*
*Benign gastroduodenal disorder*	27 (2.3)	98 (9.8)	*NS*
*Benign colonic disorder*	12 (1)	125 (12.5)	*.004*
*Esophagitis*	18 (1.5)	21 (2.1)	*NS*
*Gallbladder disease*	19 (1.6)	6 (0.6)	*NS*
*Acute viral illness*	15 (1.3)	136 (13.5)	*.005*
*Depressive disorder*	84 (7.1)	10 (1)	*NS*
*Reactive adenitis*	86 (7.3)	4 (0.4)	*NS*
*Autoimmune rheumatic disease*	40 (3.4)	7 (0.7)	*NS*
*Non-malignant lung and/or pleural disease*	46 (3.9)	8 (0.8)	*NS*
Subtotal	1009 (85.1)	885 (88.1)	
*Other diagnoses and undiagnosed cases*	177 (14.9)	119 (11.9)	
Total	1186 (100)	1004 (100)	

Data expressed as number (percentage).

*Leukemia, myelodysplastic syndrome and multiple myeloma.

PHC denotes primary care; QDU, quick diagnosis unit; ED, emergency department; *NS*, nonsignificant.

### Patients’ characteristics according to the reason for consultation

Additional file 1: Table S1 shows the main demographic and clinical characteristics and time to diagnosis of the 2 groups of patients according to the 8 main reasons for consultation. Significant differences were observed with regard to anemia: while PHC-QDU patients with anemia were older than PHC-ED-QDU patients, PHC-ED-QDU patients had lower hemoglobin concentrations, presented with more anemic syndrome and required more blood transfusions than PHC-QDU patients.

### Multivariate analysis

In the multivariate analysis, anemia (OR 2.87, 95% CI 1.49–4.55, *P < .001*), rectorrhagia (OR 2.18, 95% CI 1.10–3.77, *P = .01*) and febrile syndrome (OR 2.53, 95% CI 1.33-4.12, *P = .002*) were independent factors associated with ED referral.

## Discussion

Our results show that over 90% of patients referred from PHC to ED might have been directly referred to QDU, avoiding interposed ED visits. This causes inconvenience to patient and physician alike.

A large proportion of the increase in ED use in developed countries may be due to wasteful and inefficient inappropriate visits [14] which, one estimate suggests, may result in overuse costing $38 billion a year in the USA [15,16], with 7-89% of ED visits in different countries reportedly for non-urgent problems susceptible to less specialized care [17]. Factors influencing this situation may include inconsistent definitions of appropriateness and non-urgent triage. Indeed, such a wide range of inappropriate ED attendances underlines the difficulty in ascertaining their true rate.

A recent US study showed that the presenting complaints of patients finally diagnosed with non-urgent disorders or with more severe disorders overlap extensively, suggesting that rather than blaming patients or physicians when the disorder is identified as non-severe, greater integration of care levels should be emphasized [18]. Although most QDU patients have non-specific symptoms, making objective classification of urgent conditions difficult, we estimated that ED referrals were theoretically appropriate in a minority of patients. Although the research question of our study was primary related to referral decision-making and not the cost of care, some cost implications may be inferred. A single visit to our hospital ED, with or without basic tests, including laboratory analysis, simple x-ray and an electrocardiogram, has a raw cost to the patient of €223, compared with €117 for a QDU visit. Thus, although > 90% of study patients had public health insurance and the evaluation was free, a direct QDU referral would have avoided a virtual cost of €208,505. Nevertheless, the number of patients referred to ED form PHC with disorders potentially evaluable at QDU make up a minimal share of the total number of daily ED attendances at our hospital (roughly 0.8%) (data not shown), meaning that any such cost inferences are likely negligible.

Emergency admissions have also increased and now represent around 65% of hospital bed occupancy in England, at a cost of $17 billion [19,20]. Although yearly emergency admissions have risen by 37% in the last decade, 29% are potentially avoidable [19]. A Norwegian report found that around 20% of emergency admissions were avoidable, with other options, including next-day appointments at specialist outpatient clinics, being available [21]. In the USA, between 2003 and 2009, virtually all of the increase in hospitalizations was due to unplanned admissions from ED (17% increase), suggesting that PHC physicians were referring some of the patients they would previously have hospitalized to ED. Reportedly, US PHC physicians are increasingly relying on ED to diagnose complex patients with potentially serious conditions, rather than handling these patients themselves [22].

The wait for the first QDU visit was longer in PHC-QDU patients, mainly because PHC referrals require pre-appointment checking by the QDU physician [6], while QDU patients referred from ED are seen much more rapidly, without need for previous checking. Although these waiting time differences were statistically significant, they were probably not clinically meaningful. However, even though the mean PHC-QDU delay was only a few days, some were as long as 8 days, and 30% of PHC-QDU patients had a diagnosis of malignant neoplasm, mainly pancreatic cancer. PHC delays in the investigation and onward referral of patients with suspected cancer and of patients not recognizing or acting upon suspicious symptoms are a serious concern and may partly explain the bad outcomes of malignant disease in the United Kingdom and Denmark [23-26].

Referrals to ED were more likely in patients with anemia, rectorrhagia and febrile syndrome. Studies show that anemia and cancer are the most-recorded diagnoses in Spanish QDU patients [4,5,27,28]. Likewise, the main reasons for hospitalization for diagnostic tests in Spain are severe anemia and suspected cancer-related unintentional weight loss [4-6,27-29]. Anemia, with or without symptoms, with hemoglobin levels below 8–9 g/l, has traditionally been a criterion for admission in our hospital [4,6]. However, since the introduction of the QDU, patients with anemia referred from PHC to ED are frequently transfused and treated in the ED before safer transfer to QDU. Patients with anemia attended in ED had significantly lower hemoglobin concentrations, more anemic syndrome and greater transfusion requirements than anemic patients referred directly to QDU, suggesting that PHC physicians assessed patients referred to ED as having a more severe disease. The fact that more patients attended in ED presented febrile syndrome and rectorrhagia may be explained by a similar rationale. Although, during the study period, patients with anemia could not be transfused in the own QDU but in a “borrowed” daycare center, this limitation has been solved as of October 2013 when the unit was integrated in an internal medicine-based daycare center.

Our study has some implications. First, the appointment process for QDU patients attended in ED has been changed. Since most of these patients are appropriately referred to QDU, since April 20013, direct electronic QDU appointments are allowed. However, checking is still needed for PHC referrals. Thus, although all study patients fulfilled the QDU referral criteria, we estimated that 18% of PHC-QDU patients might have been studied in a less rapid setting. Although the goal is as many direct referrals from PHC to QDU as possible, avoiding intermediate ED referrals and delays, these referral decisions may lead to unnecessary QDU overcrowding. In order to permit direct referrals (and perhaps even PHC direct electronic appointments) without previous checking, a narrower definition of current QDU referral criteria (Table 1) may be needed. For instance, we may need to clarify that preferably “hard and fixed, > 1 cm adenopathies” and not just “adenopathies” will be evaluated, or “unintentional weight loss, after reasonably excluding a depressive disorder”.

There is an increasing need for alternatives to hospitalization, which has been exacerbated by the reductions in real healthcare spending due to the financial crisis [4,30]. Although the QDU model provides a valuable and less costly alternative for the diagnosis of a specific, but large, group of patients, the PHC referral process requires some improvement, which may be achieved following a redefinition of the QDU evaluation criteria. Although the QDU may be more suitable for public healthcare systems, the goals of reducing costs, ED overcrowding and inappropriate admissions are shared by both private and public systems [5]. In the USA, the problem of ED overuse is not only a result of PHC lack of access. A recent report found that ED are being increasingly used by PHC physicians to do quick diagnostic studies of patients with potentially serious conditions. The report indicates that ED have direct, immediate access to advanced diagnostic tools such as magnetic resonance imaging, CT scans and nuclear scans, which are seldom accessible elsewhere or would take much longer if ordered from an outpatient setting [22].

### Limitations

The study limitations include the fact that it took place in a single center. However, the unit received patients from 12 PHC centers and the population attended is representative of that of other Spanish QDU, according to published reports [27-29,31,32]. In addition, although the appropriateness of PHC referrals to QDU and ED was objectively assessed using a scoring system, the possibility of some perception bias cannot be excluded. It can also be argued that review and scoring of medical records by somewhat junior hospital physicians (i.e., senior residents) is exposed to bias and that a better approach would have been to incorporate a PHC physician on the review group. Likewise, the possibility of some subjective assessment by the QDU attending physician at the time of scoring patients who might have been evaluated in a non-QDU setting cannot be ruled out either. Furthermore, the retrospective design represents a methodological concern because only patients who ended up in the QDU were evaluated, thus limiting the conclusions that can be drawn about the appropriateness of referrals. For instance, although the PHC-ED-QDU group did not include patients referred from PHC to ED who were then hospitalized or discharged after treatment, it is possible that a substantial proportion of such patients were properly referred to ED. Finally, our study did not address the question of why physicians who referred patients to ED decided not to use the QDU instead. Presumably, physicians who referred patients to ED also had the QDU as an option. Since the referring PHC physicians were given a list of conditions for which QDU referral was appropriate, and all patients referred to QDU indeed fulfilled the evaluation criteria, some physicians may have: 1) made the decision to refer to ED and not QDU based on their judgment of the patient condition; 2) had more confidence in ED than QDU; or 3) were unaware or had no clear knowledge of QDU referral criteria. In any way, it is imperative to bear in mind the PHC physicians’ perspectives, as these physicians may find themselves faced with a challenging clinical puzzle that they may not have the skills or time to solve and even delays of some days to have the patient evaluated may be discouraging. Similarly, there may be logical justifications why a PHC physician referred a patient to ED at the time of patient encounter that cannot be determined by a retrospective review of medical records and elements such as social factors, patient choices and circumstances, or even time of day, might all have had some effect. Therefore, we cannot infer from our results that those 93% of patients who might have been directly referred to QDU instead of ED represented “incorrect” decisions; the PHC physician’s decision-making process would actually require additional investigation. A stronger methodology through a prospective study in which, for instance, a PHC physician is promptly contacted anytime he or she refers a patient meeting QDU referral criteria to ED and the reason why he or she has made the decision is discussed, would be helpful to appreciate his or her decision-making process. A future such study evaluating this process, perhaps including also an assessment of physicians’ attitudes and aptitudes, could be valuable.

## Conclusion

In summary, over 90% of patients who were referred to ED from 12 PHC centers might have been directly referred to QDU, avoiding the inconvenience of the ED visit. In contrast, most patients referred to QDU first were appropriately referred to it. Anemia, rectorrhagia and febrile syndrome were independent factors associated with ED referral. Although it may seem paradoxical that the mean wait for the first QDU visit was longer in PHC-QDU than in PHC-ED-QDU patients, this is mainly explained by the fact that PHC-QDU referrals require pre-appointment checking by the QDU physician, while patients referred from ED do not. Since we also found that nearly one-fifth of all QDU patients might have been studied in a less rapid, less acute, non-QDU setting, a narrower definition of QDU evaluation criteria may be needed to improve and hasten referrals from PHC.

## Competing interests

The authors declare that they have no competing interests.

## Authors’ contributions

XB drafted the manuscript, coordinated the study, participated in the conception and design of the study, acquisition of data and statistical analysis, OE participated in the acquisition of data and statistical analysis. DN participated in the acquisition of data. EC participated in the acquisition of data and statistical analysis. SF participated in the acquisition of data. AC helped to draft the manuscript. ALS participated in the conception and design of the study and helped to draft the manuscript. All authors participated in the analysis and interpretation of data, revised the manuscript for intellectual content and read and approved the final manuscript.

## Authors’ information

Xavier Bosch is the QDU attending physician at the Department of Internal Medicine. Ona Escoda, David Nicolás, Emmanuel Coloma and Sara Fernández are residents at the Department of Internal Medicine. Antonio Coca is the director of the Clinical Institute of Medicine and Dermatology (ICMiD). Alfonso López-Soto is the head of the Department of Internal Medicine. All the authors are affiliated with the Hospital Clínic and the University of Barcelona.

## Pre-publication history

The pre-publication history for this paper can be accessed here:

http://www.biomedcentral.com/1471-2296/15/75/prepub

## Supplementary Material

Additional file 1: Table S1 Main characteristics and differences of the two groups of patients according to the eight main reasons for consultation. Data expressed as mean (SD) and median [25th-75th percentiles] or number (percentage). PHC denotes primary care; QDU, quick diagnosis unit; ED, emergency department; Charlson com in., Charlson comorbidity index; GI, gastrointestinal; *NS*, nonsignificant; IBS, irritable bowel syndrome; CLD, chronic liver disease. Click here for file

## References

[B1] Pines JM Hilton JA Weber EJ Alkemade AJ Al Shabanah H Anderson PD Bernhard M Bertini A Gries A Ferrandiz S Kumar VA Harjola VP Hogan B Madsen B Mason S Ohlén G Rainer T Rathlev N Revue E Richardson D Sattarian M Schull MJ International perspectives on emergency department crowding Acad Emerg Med 2011 18 1358 1370 22168200 10.1111/j.1553-2712.2011.01235.x

[B2] Miró O State of emergency medicine in Spain Int J Emerg Med 2010 3 219 226 21373287 10.1007/s12245-010-0249-x PMC3047875

[B3] Akbari A Mayhew A Al-Alawi MA Grimshaw J Winkens R Glidewell E Pritchard C Thomas R Fraser C Interventions to improve outpatient referrals from primary care to secondary care Cochrane Database Syst Rev 2008 4 CD005471 10.1002/14651858.CD005471.pub2 PMC4164370 18843691

[B4] Pericás JM Aibar J Soler N López-Soto A Sanclemente-Ansó C Bosch X Should alternatives to conventional hospitalisation be promoted in an era of financial constraint? Eur J Clin Invest 2013 43 602 615 23590593 10.1111/eci.12087

[B5] Bosch X Moreno P Ríos M Jordán A López-Soto A Comparison of quick diagnosis units and conventional hospitalization for the diagnosis of cancer in Spain: a descriptive cohort study Oncology 2012 83 283 291 22964843 10.1159/000341658

[B6] Bosch X Jordán A Lopez-Soto A Quick diagnosis units: avoiding referrals from primary care to the ED and hospitalizations Am J Emerg Med 2013 31 114 123 22980360 10.1016/j.ajem.2012.06.013

[B7] World Health Organization International Classification of Diseases (ICD). 10th Revision (ICD-10) 2014 Available at: http://www.who.int/classifications/icd/en Accessed February 28, 2014

[B8] Knaus WA Draper EA Wagner DP Zimmerman JE APACHE II: a severity of disease classification system Crit Care Med 1985 13 818 829 3928249

[B9] Turk DC Rudy TE Sorkin BA Neglected topics in chronic pain treatment outcome studies: determination of success Pain 1993 53 3 16 8316386 10.1016/0304-3959(93)90049-U

[B10] Charlson ME Pompei P Ales KL MacKenzie CR A new method of classifying prognostic comorbidity in longitudinal studies: development and validation J Chron Dis 1987 40 373 383 3558716 10.1016/0021-9681(87)90171-8

[B11] Ware JE Jr Sherbourne CD The MOS 36-item short-form health survey (SF-36). I. Conceptual framework and item selection Med Care 1992 30 473 483 1593914

[B12] van der Putten JJ Hobart JC Freeman JA Thompson AJ Measuring change in disability after inpatient rehabilitation: comparison of the responsiveness of the Barthel index and the Functional Independence Measure J Neurol Neurosurg Psychiatry 1999 66 480 484 10201420 10.1136/jnnp.66.4.480 PMC1736299

[B13] Altman DG Practical Statistics for Medical Research 1991 London: Chapman & Hall 403 409

[B14] Flores-Mateo G Violan-Fors C Carrillo-Santisteve P Peiró S Argimon JM Effectiveness of organizational interventions to reduce emergency department utilization: a systematic review PLoS One 2012 7 e35903 22567118 10.1371/journal.pone.0035903 PMC3342316

[B15] Adams JG Emergency department overuse: perceptions and solutions JAMA 2013 309 1173 1174 23512065 10.1001/jama.2013.2476

[B16] NEHI Research Brief A Matter of Urgency: Reducing Emergency Department Overuse 2010 Available at: http://www.nehi.net/writable/publication_files/file/nehi_ed_overuse_issue_brief_032610finaledits.pdf Accessed February 28, 2014

[B17] Khangura JK Flodgren G Perera R Rowe BH Shepperd S Primary care professionals providing non-urgent care in hospital emergency departments Cochrane Database Syst Rev 2012 11 CD002097 10.1002/14651858.CD002097.pub3 PMC4164956 23152213

[B18] Raven MC Lowe RA Maselli J Hsia RY Comparison of presenting complaint vs discharge diagnosis for identifying “nonemergency" emergency department visits JAMA 2013 309 1145 1153 23512061 10.1001/jama.2013.1948 PMC3711676

[B19] Nagpaul C Should GPs be fined for rises in avoidable emergency admissions to hospital? No BMJ 2013 346 f1391 10.1136/bmj.f1391 23463140

[B20] Blunt I Bardsley M Dixon J Trends in Emergency Admissions in England 2004–2009 2010 The Nuffield Trust Available at: http://www.nuffieldtrust.org.uk/publications/trends-emergency-admissions-england-2004-2009 Accessed February 28, 2014.

[B21] Lillebo B Dyrstad B Grimsmo A Avoidable emergency admissions? Emerg Med J 2013 30 707 711 22983980 10.1136/emermed-2012-201630

[B22] Gonzalez Morganti K Bauhoff S Blanchard JC Abir M Iyer N Smith AC Vesely JV Okeke EN Kellermann AL The Evolving Role of Emergency Departments in the United States 2013 Available at: http://www.rand.org/pubs/research_reports/RR280.html Accessed February 28, 2014 10.7249/rhq-03-02-03 PMC4945168 28083290

[B23] Neal RD Do diagnostic delays in cancer matter? Br J Cancer 2009 101 suppl 2 S9 S12 19956171 10.1038/sj.bjc.6605384 PMC2790696

[B24] Hansen RP Vedsted P Sokolowski I Søndergaard J Olesen F Time intervals from first symptom to treatment of cancer: a cohort study of 2,212 newly diagnosed cancer patients BMC Health Serv Res 2011 11 284 22027084 10.1186/1472-6963-11-284 PMC3217887

[B25] Abdel-Rahman M Stockton D Rachet B Hakulinen T Coleman MP What if cancer survival in Britain were the same as in Europe: how many deaths are avoidable? Br J Cancer 2009 101 S115 S124 19956155 10.1038/sj.bjc.6605401 PMC2790713

[B26] Christensen KG Fenger-Grøn M Flarup KR Vedsted P Use of general practice, diagnostic investigations and hospital services before and after cancer diagnosis - a population-based nationwide registry study of 127,000 incident adult cancer patients BMC Health Serv Res 2012 12 224 22838741 10.1186/1472-6963-12-224 PMC3507912

[B27] Capell S Comas P Piella T Rigau J Pruna X Martínez F Montull S Quick and early diagnostic outpatient unit: an effective and efficient assistential model. Five years experience Med Clin (Barc) 2004 123 247 250 15482729 10.1016/s0025-7753(04)74478-4

[B28] Rubio-Rivas M Vidaller A Pujol i Farriols R Mast R Rapid diagnosis unit in a third level hospital. Descriptive study of the first year and a half Rev Clin Esp 2008 208 561 563 19121267 10.1016/s0014-2565(08)76034-x

[B29] Bosch X Aíbar J Capell S Coca A López-Soto A Quick diagnosis units: a potentially useful alternative to conventional hospitalisation Med J Aust 2009 191 496 498 19883344 10.5694/j.1326-5377.2009.tb02912.x

[B30] Organisation for Economic Co-operation and Development Health at a Glance: Europe 2012 2012 OECD Publishing Available at: http://dx.doi.org/10.1787/9789264183896-en Accessed February 28, 2014

[B31] De Santos Castro PA Jimeno Cargues A García Cobo MC Study on the immediate care clinics of the internal medicine department (University Clinic Hospital of Valladolid) Rev Clin Esp 2006 206 84 89 16527167 10.1157/13085358

[B32] Torres M Capdevila JA Armario P Montull S Conventional hospitalization alternatives in internal medicine Med Clin (Barc) 2005 124 620 626 15871780 10.1157/13074393

