# Supplementary material for: Primary care referrals of patients with potentially serious diseases to the emergency department or a quick diagnosis unit: a cross-sectional retrospective study
Source: BMC Fam Pract. 2014 Apr 28;15:75. doi: 10.1186/1471-2296-15-75 (PMC4021313; doi:10.1186/1471-2296-15-75)
Supplement: Additional file 1: Table S1 — Main characteristics and differences of the two groups of patients according to the eight main reasons for consultation. Data expressed as mean (SD) and median [25th-75th percentiles] or number (percentage). PHC denotes primary care; QDU, quick diagnosis unit; ED, emergency department; Charlson com in., Charlson comorbidity index; GI, gastrointestinal; NS, nonsignificant; IBS, irritable bowel syndrome; CLD, chronic liver disease. [file 1471-2296-15-75-S1.docx]

**Additional file 1: Table S1*.* Main characteristics and differences of the two groups of patients according to the eight main reasons for consultation**

| **Anemia** | **PHC-QDU Patients**  **(n=195)** | **PHC-ED-QDU Patients**  **(n=283)** | ***P* value** |
| --- | --- | --- | --- |
| Age (years) | 74.4 (18.3), 77.5 [68.1;83.3] | 52.1 (12.9), 56 [49.6;61.4] | ***< .0001*** |
| Female | 107 (54.7%) | 151 (53.2%) |  |
| Male | 88 (45.3%) | 132 (46.8%) |  |
| Time to diagnosis (days) | 7.9 (1.6), 7.3 [6.7;9.4] | 8.3 (3.7), 8 [7.4;9.9] | *NS* |
| Hemoglobin level (g/L) | 88.8 (26.7), 89.8 [85.3;89.9] | 76.7 (19.8), 77.9 [73;78.2] | ***.03*** |
| Anemic syndrome | 128 (65.6%) | 231 (81.6%) | ***< .001*** |
| Transfusion | 36 (18.5%) | 86 (30.4%) | ***.004*** |
| Charlson com in. (score) | 1.1 (0.8), 1 [0.8;1.3] | 1.4 (1), 1.2 [1;1.5] | *NS* |
| Main diagnosis | Iron-deficiency anemia/Colorectal cancer | Iron-deficiency anemia/Benign GI disease |  |
| **Unintentional weight loss** | **PHC-QDU Patients (n=293)** | **PHC-ED-QDU Patients (n=123)** | ***P* value** |
| Age (years) | 63.4 (13.5), 66.5 [60;76.5] | 65.7 (15), 70.5 [62;79.5] | *NS* |
| Female | 146 (49.8%) | 60 (48.6%) |  |
| Male | 147 (50.2%) | 63 (51.4%) |  |
| Time to diagnosis (days) | 10.2 (3.2), 9.8 [8.5;12.2] | 10.5 (4.1), 10.1 [9.2;12.5] | *NS* |
| Charlson com in. (score) | 1.4 (0.9), 1.3 [1.2;1.5] | 1.3 (0.8), 1.1 [1;1.4] | *NS* |
| Main diagnosis | Pancreatic cancer/ Depressive disorder | Pancreatic cancer |  |
| **Febrile Syndrome** | **PHC-QDU Patients (n=135)** | **PHC-ED-QDU Patients (n=237)** | ***P* value** |
| Age (years) | 49.6 (13.7), 52 [43.5;58] | 44.3 (11.1), 46.8 [40;52.1] | *NS* |
| Female | 70 (52%) | 127 (53.5%) |  |
| Male | 65 (48%) | 110 (46.5%) |  |
| Time to diagnosis (days) | 11.1 (6.5), 10.5 [9.3;12.4] | 9.1 (3.4), 8.3 [8;11.2] | *NS* |
| Charlson com in. (score) | 1.1 (0.8), 1 [0.8;1.3] | 1.2 (0.7), 0.9 [0.7;1.2] | *NS* |
| Main diagnosis | Lymphoma | Acute viral illness |  |
| **Adenopathies and/or Palpable Masses** | **PHC-QDU Patients (n=149)** | **PHC-ED-QDU Patients (n=37)** | ***P* value** |
| Age (years) | 52.3 (14.8), 56 [49;61.4] | 60.1 (16.5), 63 [56.3;69] | *NS* |
| Female | 79 (53%) | 19 (50.2%) |  |
| Male | 70 (47%) | 18 (49.8%) |  |
| Time to diagnosis (days) | 9.8 (5.6), 9.1 [8.9;11] | 8.2 (3.3), 8 [7.8;9.5] | *NS* |
| Charlson com in. (score) | 1.2 (0.7), 1 [0.7;1.2] | 1.2 (0.8), 1.1 [0.8;1.4] | *NS* |
| Main diagnosis | Lymphoma/ Reactive adenitis | Lymphoma |  |
| **Lung and/or Pleural Abnormalities** | **PHC-QDU Patients (n=66)** | **PHC-ED-QDU Patients (n=31)** | ***P* value** |
| Age (years) | 60.2 (16.3), 63 [57.5;67] | 62.5 (17.6), 64.5 [58;69.2] | *NS* |
| Female | 25 (37.9%) | 11 (35.5%) |  |
| Male | 41 (62.1%) | 20 (64.5%) |  |
| Time to diagnosis (days) | 9.9 (7), 9.5 [8;11.2] | 10.2 (8), 9.8 [8.2;11.4] | *NS* |
| Charlson com in. (score) | 1.3 (0.9), 1.2 [1;1.6] | 1.2 (0.6), 1.1 [0.9;1.5] | *NS* |
| Main diagnosis | Lung cancer | Lung cancer |  |
| **Chronic Diarrhea** | **PHC-QDU Patients (n=52)** | **PHC-ED-QDU Patients (n=22)** | ***P* value** |
| Age (years) | 51.2 (16.3), 54 [48;59.3] | 53.3 (17.8), 58 [52.3;63] | *NS* |
| Female | 27 (51.9%) | 12 (54.5%) |  |
| Male | 25 (48.1%) | 10 (45.5%) |  |
| Time to diagnosis (days) | 10.2 (3.8), 9.4 [9;12.2] | 10 (3.2), 9.1 [8.6;11.6] | *NS* |
| Charlson com in. (score) | 1.3 (0.9), 1.1 [1;1.5] | 1.2 (0.7), 0.9 [0.7;1.4] | *NS* |
| Main diagnosis | IBS | IBS |  |
| **Ascites** | **PHC-QDU Patients (n=26)** | **PHC-ED-QDU Patients (n=21)** | ***P* value** |
| Age (years) | 66.2 (18.3), 69 [63;73.5] | 65.5 (17), 68.5 [62.5;73] | *NS* |
| Female | 15 (57%) | 12 (55.5%) |  |
| Male | 11 (43%) | 9 (44.5%) |  |
| Time to diagnosis (days) | 7.3 (2.3), 6.8 [6.3;8.9] | 7.1 (2), 6.6 [6.2;8.7] | *NS* |
| Charlson com in. (score) | 1.3 (1), 1.1 [0.9;1.5] | 1.1 (0.5), 1 [0.8;1.4] | *NS* |
| Main diagnoses | Malignant ascites/CLD | Malignant ascites |  |
| **Rectorrhagia** | **PHC-QDU Patients (n=44)** | **PHC-ED-QDU Patients (n=163)** | ***P* value** |
| Age (years) | 53.1 (17.2), 57.5 [51.2;62.5] | 51 (16), 55 [50;60.7] | *NS* |
| Female | 23 (53%) | 89 (54.6%) |  |
| Male | 21 (47%) | 74 (45.4%) |  |
| Time to diagnosis (days) | 7.5 (2.1), 6.7 [6.1;8.2] | 7.9 (4), 7.4 [6.6;8.3] | *NS* |
| Charlson com in. (score) | 1.4 (1), 1.3 [1;1.4] | 1.3 (0.7), 1.1 [0.8;1.3] | *NS* |
| Main diagnosis | Colorectal cancer | Benign colonic disorder |  |

Data expressed as mean (SD) and median [25^th^-75^th^ percentiles] or number (percentage)

PHC denotes primary care; QDU, quick diagnosis unit; ED, emergency department; Charlson com in., Charlson comorbidity index; GI, gastrointestinal; *NS*, nonsignificant; IBS, irritable bowel syndrome; CLD, chronic liver disease
